# In Search of Epigenetic Marks in Testes and Sperm Cells of Differentially Fed Boars

**DOI:** 10.1371/journal.pone.0078691

**Published:** 2013-11-04

**Authors:** Rémy Bruggmann, Vidhya Jagannathan, Martin Braunschweig

**Affiliations:** 1 Department of Biology, Bioinformatics, University of Bern, Berne, Switzerland; 2 Institute of Genetics, Vetsuisse Faculty, University of Bern, Berne, Switzerland; Victor Chang Cardiac Research Institute, Australia

## Abstract

In search of transmittable epigenetic marks we investigated gene expression in testes and sperm cells of differentially fed F0 boars from a three generation pig feeding experiment that showed phenotypic differences in the F2 generation. RNA samples from 8 testes of boars that received either a diet enriched in methylating micronutrients or a control diet were analyzed by microarray analysis. We found moderate differential expression between testes of differentially fed boars with a high FDR of 0.82 indicating that most of the differentially expressed genes were false positives. Nevertheless, we performed a pathway analysis and found disparate pathway maps of development_A2B receptor: action via G-protein alpha s, cell adhesion_Tight junctions and cell adhesion_Endothelial cell contacts by junctional mechanisms which show inconclusive relation to epigenetic inheritance. Four RNA samples from sperm cells of these differentially fed boars were analyzed by RNA-Seq methodology. We found no differential gene expression in sperm cells of the two groups (adjusted *P*-value>0.05). Nevertheless, we also explored gene expression in sperm by a pathway analysis showing that genes were enriched for the pathway maps of bacterial infections in cystic fibrosis (CF) airways, glycolysis and gluconeogenesis p.3 and cell cycle_Initiation of mitosis. Again, these pathway maps are miscellaneous without an obvious relationship to epigenetic inheritance. It is concluded that the methylating micronutrients moderately if at all affects RNA expression in testes of differentially fed boars. Furthermore, gene expression in sperm cells is not significantly affected by extensive supplementation of methylating micronutrients and thus RNA molecules could not be established as the epigenetic mark in this feeding experiment.

## Introduction

Transgenerational epigenetic inheritance in mammals is the transmission of environmentally induced epigenetic states to progeny that were not exposed to that environment including the germ cells from which they come from [1]. The extent and the specificity to which acquired epigenetic modifications during an individual's life time are transmitted to next generations are controversially discussed. However, effects of environmental exposure on pregnant females that are reflected in the subsequent F1 and F2 generations are also considered as transgenerational epigenetic inheritance [2]. These authors further introduced the term ‘transgenerational epigenetic inheritance via the gametes’ that refers to effects on the phenotype that are non-mendelian and transmitted as epigenetic marks including RNA and proteins via the gametes. It is well established in mice that the epigenome undergoes genome wide epigenetic reprogramming during gametogenesis and embryogenesis [3], [4]. It is assumed that this epigenetic resetting is conserved in mammals arguing against widespread inheritance of epigenetic modifications induced by ancestral environmental effects. However, there are chromosomal regions that are particularly resistant to reprogramming [5]. The extensively studied and often quoted examples of inducible DNA methylation in IAP retrotransposons at the agouti viable yellow (*A^yv^*) and the axin fused (Axin*^Fu^*) alleles did not persist to the third generation [6], [7]. Transgenerational effects were demonstrated in an epidemiological study in humans from Northern Sweden. These Överkalix data showed a link between grandparental food supply during their slow growth period and the mortality risk ratio of their grandsons [8]. More recently it was reported that F1 offspring responded to F0 paternal protein diet showing elevated hepatic expression of many genes that are involved in lipid and cholesterol biosynthesis in the low-protein diet compared to the control group. In addition reproducible changes in DNA methylation were detected at a putative enhancer of the lipid regulator *Ppara* [9]. Transgenerational perpetuation of DNA methylation was assumed to be a result of incomplete DNA methylation erasure during gametogenesis and early embryogenesis. At least at the *A^vy^* allele it was shown that DNA methylation is unlikely to be the epigenetic mark that transmits the pseudoagouti phenotype [10]. A paramutation-like phenomenon was described in progeny of heterozygotes *Kit^tm1Alf/+^* mice [11]. The *tm1Alf* mutation abrogates the synthesis of Kit tyrosine kinase receptor and wild type progeny from heterozygous *Kit^tm1Alf/+^* parents showed the mutant phenotype of a white tail dip and white feet. Furthermore, these wild type mice transmitted the white tail dip and white feet mutant phenotype to subsequent generations in the absence of a *tm1Alf* allele. A similar demonstration of paramutation in mice was achieved by microinjecting microRNA miR-1 in fertilized oocytes to target the Cdk9 cardiac growth regulator [12]. Mice born after miR-1 injection showed an increased heart size compared to controls and this phenotype was transmitted to next generations. These results suggest that RNA molecules play a role in non-mendelian inheritance. Transgenerational epigenetic inheritance attracted much attention because it is challenging the paradigm that solely DNA transmits all the information for subsequent living organisms. Irrespective of a small number of particular examples the relevance of transgenerational epigenetic inheritance characterized by epigenetic marks that are persisting over generations is widely unknown.

Recently we presented data from a three generation pig feeding experiment by which we show a transgenerational response in F2 offspring [13]. F0 boars were fed a diet enriched in methylating micronutrients or a control diet. In the F2 descendants from these boars we found significant gene expression, carcass and DNA methylation differences. It is hypothesized that if non-mendelian inheritance took place in this three generation pig pedigree then the information could only be transmitted via the F0 boars' semen because exclusively the F0 boars were differentially fed in this pedigree. We suggested studying testes sample because potential differences in gene expression could also point towards other epigenetic marks than RNA molecules. From the analysis of RNA in sperm cells we expected to find differences of gene expression that are directly carried to the egg via fertilization and would eventually be involved in early embryogenesis. These potential changes must then be maintained in the germ cells of the F1 generation to be effective in a F2 generation. In the present study we analyzed RNA microarray data from testes and RNA-Seq data from sperm cells of these differentially fed F0 boars in search of segregating epigenetic RNA marks.

## Results

### Gene Expression Profiling in testes by microarray analysis

Gene expression was investigated by microarray gene expression profiling to compare gene expression levels in testes of 4 F0 boars that received from month one to month ten an experimental diet enriched in methylating micronutrients with those 4 boars that received a control diet. From the total of represented 43,663 probes on the porcine microarray chip 35,285 showed signals and 31,262 probes were with non-negligible variation (detection *P*<0.05). A numerical overview of the gene expression analysis is given in Table? 1. Note that the false discovery rate (FDR) is 0.82 and thus considerably high. We found two fold differences in mRNA levels in F0 testes between the two groups for 8 genes (*P*<0.01) and a subtle≥one fold change for 70 genes (*P*<0.01).

**Table 1:** Probe counts by significance and fold-change (fc) in testes of F0 boars.

*P*-value	#significants	FDR	fc≥1		fc≥1.5	fc≥2	fc≥3	fc≥4	fc≥8
*<*0.1	1583	0.82	1583	331		52	5	2	0
*P<*0.01	70	0.82	70	29		8	2	1	0
*P<*0.001	9	0.82	9	5		1	0	0	0
*P<*1e-04	1	0.82	1	1		1	0	0	0
*P<*1e-05	0	NA	0	0		0	0	0	0

#significants: Number of significant probes that differ between the two groups on the significance level indicated.

FDR: False discovery rate.

### Pathway analysis of differentially expressed genes in testes

Although the FDR of the differential gene expression was 0.82 indicating that most of the differentially expressed genes might be false positives we performed a pathway analysis in order not to miss any hint of transmissible epigenetic marks. From 659 features that indicated differential expression between the two groups (*P*<0.05, FDR = 0.82) 526 could be annotated and 482 were suitable for the enrichment analysis (Table S1). The largest number of differentially expressed genes between the two diet groups was associated with pathway maps of development_A2B receptor: action via G-protein alpha s, Cell adhesion_Tight junctions and Cell adhesion_Endothelial cell contacts by junctional mechanisms. The gene ontology (GO) processes that match most of the gene expression data are positive regulation of nucleobase-containing compound metabolic process, cellular response to hormone stimulus and cellular process. A summary of the enrichment analysis is given in Table S2. The *P*-values of the pathway analysis are biased because they are based on the gene list from the gene expression analysis (FDR = 0.82).

### RNA in sperm cells of differentially fed F0 boars analysed by RNA-Seq

An RNA-Seq experiment was performed in order to comprehensively investigate if there are differences in the expression of RNA molecules>100 nt in sperm cells of differentially fed F0 boars. From this experiment we obtained between 27.5 million and 42.5 million reads for each of the respective sperm RNA sample from 4 differentially fed boars (Table? 2). After mapping, removing of unambiguous hits and removing of PCR duplicates we ended up with 4,454,674 and 2,832,484 mapped reads for the two boars that received the methyl supplemented diet and with 3,069,580 and 1,794,219 reads for the two boars that received the control diet, respectively. These numbers of reads were compared in a DESeq analysis to search for differences in the expression of RNA transcripts in sperm cells between the two groups. The overall DESeq analysis showed no significant differences between the two groups in RNA expression based on the *P*-value adjusted for multiple testing (Table S4). However, we could annotate 105 genes that were differently expressed between the two diet groups on the nominal *P*-value<0.05 (Table S4). Although not significant based on the adjusted *P*-value we performed an enrichment analysis in order not to miss any clue of an epigenetic mark. We found that the largest portion of these differentially expressed genes was enriched for the pathway maps of bacterial infections in cystic fibrosis (CF) airways, glycolysis and gluconeogenesis p.3 and cell cycle_Initiation of mitosis. The GO processes including the most of these differentially expressed genes were viral transcription and viral genome expression, viral infectious cycle, cellular protein localization, cellular macromolecule localization, nuclear-transcribed mRNA catabolic process, nonsense-mediated decay and cellular component organization at cellular level. A summary of the enrichment analysis of sperm RNA from differentially fed boars is presented in Table S3. Again, the *P*-values of the pathway analysis are based on the gene list from the gene expression analysis which does not reveal significant expression differences when corrected for multiple testing (*P*-value adjusted).

**Table 2:** Mapping statistics of illumina reads.

Sample ID	# reads	# mapped	[%]	# unmapped	[%]	# ambiguous	[%]	# unique	[%]	# no PCR-	[%]
										duplicates	
9597 suppl. diet	42,532,268	39,498,715	92.9	3,033,553	7.1	24,384,633	57.3	15,114,082	35.5	4,454,674	10.5
9598 control diet	34,536,286	31,908,341	92.4	2,627,945	7.6	17,195,879	49.8	14,712,462	42.6	3,069,580	8.9
9599 suppl. diet	39,129,530	36,094,046	92.2	3,035,484	7.8	22,057,905	56.4	14,036,141	35.9	2,832,484	7.2
9600 control diet	27,527,320	25,759,633	93.6	1,767,687	6.4	15,499,187	56.3	10,260,446	37.3	1,794,219	6.5

## Discussion

We investigated RNA samples from differentially fed F0 boars from a three generation pedigree that showed a transgenerational epigenetic response in the F2 offspring. Exclusively the experimental F0 boars received high doses of methylating micronutrients intending to unbalance the one-carbon metabolism [14].

We hypothesized that differential gene expression in testes and sperm cells of differentially fed F0 boars is prerequisite to find epigenetic RNA marks. We found 482 differentially expressed genes (P<0.05) between the two groups of testes samples for which the FDR was considerably high for all transcripts (FDR =  0.82) and thus includes a high portion of false positives. A two fold difference in expression was found for 8 transcripts (FDR = 0.82). We consider the gene expression differences between the two groups of 4 boars as moderate. The pathway maps and GO processes associated with gene expression differences do not indicate a simple relationship between nutritional influences and gene expression in testes which may reflect the high FDR of the involved differentially expressed genes. Nevertheless the Adenosine A2B receptor influences cell differentiation and proliferation and has thus far reaching consequences (http://host.genego.com/map_482.php). The positive regulation of nucleobase-containing compound metabolic process, the cellular response to hormone stimulus and the cellular process represent also complex networks making it difficult to interpret them in the light of epigenetic inheritance. The expression result is thus not conclusive of whether the diet affects processes related to transmittable epigenetic marks. The results, however, indicate that the extreme supplementation of methylating micronutrients from month one to month ten of age has a very moderate (if any) effect on gene expression in boar testes as measured by microarray analysis.

A more direct way in search of epigenetic marks was the RNASeq experiment of sperm RNA between groups of each of two differentially fed F0 boars. According to the adjusted *P*-value there was no difference in RNA expression of sizes larger than 100 nt. Consequently, it is concluded that RNA expression in sperm cells is not significantly affected by extensive supplementation of methylating micronutrients and thus RNA could not be established as epigenetic mark in this feeding experiment. Furthermore, it is emphasized that potential epigenetic marks or their epigenetic effects in the F0 generation must persists up to the sperm cells of the F1 generation to be considered causal for observed effects in the F2 generation. The sample size in this study was rather small and only pronounced differences in RNA expression would have been detected. There are following possibilities to interpret the data: (1) The diet does not induce gene expression differences that can be reliably measured in testes or sperm cells. (2) The diet induces epigenetic modifications but RNA molecules larger than 100 nt are not the epigenetic marks. (3) The diet induces epigenetic modifications and RNA molecules larger than 100 nt are differentially expressed but it could not be demonstrated in this study because of small sample size and/or small expression differences. In mammals, there is still no experimental evidence if transgenerational epigenetic inheritance in the narrow sense is contributing to the variation of multifactorial traits including diseases challenging its relevance [15].

## Conclusion

We did not find conclusive evidence that high dose of methylating micronutrients significantly affect the expression of RNA molecules longer than 100 nt in testes or sperm cells of boars. Our experiment suggests that RNA molecules larger than 100 nt in sperm cell formation is well protected against nutritional influences.

## Materials and Methods

### Ethics Statement

The study was carried out in strict accordance with Swiss Federal Law on Animal Protection of 16 December 2005 (Tierschutzgesetz TSchG, SR 455), Art. 32, Absatz 1; Ordinance on Animal Protection of 23 April 2008 (Tierschutzverordnung TSchV, SR 455.1). Approval was not necessary since the experiment was considered to cause no harm to the experimental animals (Art. 62 Abs. 1 TSchV, degree of severity 0).

### Animals and diet

Boars from the Large White breed were kept in a research facility at Agroscope Liebefeld-Posieux Research Station ALP, which employs a veterinarian for the health care of the experimental animals, and which is surveyed by the welfare department of the canton of Fribourg (Switzerland). One group of 8 boars received from one month of age until slaughtering at 10 months of age an experimental diet enriched in methylating micronutrients and the other group of boars were fed a control diet. The composition of the methylating micronutrients that were added to the diet is given in Table? 3. The boars were sacrificed by a certified butcher using a captive bolt device. The testes samples used for the microarray analysis were from 8 boars of three litters born from 3 sows mated to 2 boars. These boars were randomly allotted within litter to 2 feeding groups. For the RNASeq experiment we used semen samples from one of the above litter of 4 full brothers.

**Table 3:** Methylating nutrients contents of the diets fed the F0 boars (per kg diet).

	Control diet				Experimental diet			
Age, months	Starter 1–2.5	Grower 2.5–4	Finisher 4–5	Boar 5–10	Starter 1–2.5	Grower 2.5–4	Finisher 4–5	Boar 5–10
Methionine, g	4.4	3.3	2.5	3.3	11.6	8.5	6.6	8.5
Cysteine, g	3.3	3.5	3.2	2.9	3.2	3.4	3.2	2.9
Choline, mg	300	200			1300	1400		
Betaine, mg	0	0			1600	1600		
Vit. B6, mg	4	3			1600	1600		
Folate, mg	0.5	0.5			200	200		
Vit. B12, mg	0.02	0.02			8	8		

### Microarray expression analysis

The microarray analysis was performed as described by Braunschweig et al. [13]. RNA from testes tissue samples was extracted using the Trizol reagent according to the manufacturer's protocol. The integrity of RNA was confirmed by a Bioanalyzer 2100 (Agilent, Waldbronn, Germany). The RNA was labeled and hybridized to the porcine gene expression microarray from Agilent Technologies according to standard protocol used at the Functional Genomic Center Zürich. We used the Porcine (V2) Gene Expression Microarray, 4644K (G2519F). Spot intensities that were obtained from the hybridization of the samples to the probes were extracted from the TIFF images using Agilent Feature Extraction Software 9.5. From the generated TXT files the ‘‘gMedianSignal’’ of the spots was used as raw expression value and further analyzed using R/Bioconductor. A signal of probe was declared present in a condition if it had a linear signal value above 25 and if the flag ‘‘gIsWellAboveBG’’ generated by the Feature Extraction software was true in at least 50% of the replicates of that condition. False Discovery Rates were computed using the Benjamini-Hochberg method. We used 4 testes tissue samples from F0 boars that received the diet enriched in methylation micronutrients and 4 samples from the control boar group.

All probes from the microarray experiment that had a *P*-value less than 0.05 for the difference between signal averages of the two groups were manually annotate and analyzed using the GeneGO MetaCore pathway analysis software (db version 6.2, build 24095, http://www.genego.com/metacore.php). The software interconnected all candidate genes according to published literature-based annotations. Only direct connections between the identified genes were considered. In MetaCore analysis, the statistical significance of networks is indicated by a P-value from the Fisher's exact test. The false discovery rate (FDR) is used for multiple testing corrections.

### RNA from sperm cells

Boar sperm cells were obtained by flushing epididymis with semen extender, frozen in liquid nitrogen and stored at −80°C. The semen was washed twice with somatic cell lysis buffer (0.1% SDS, 0.5% Triton X-100 in dH2O). Sperm cells were pelleted and resuspended in 2 ml Trizol reagent. The mixture was homogenized by lysing cells using more than 30 strokes with a 26-gauge needle. RNA was then extracted following the manufacture's protocol. The integrity of the RNA was tested on a Bioanalyzer 2100 (Agilent, Waldbronn, Germany). Sperm RNA was directly used for library construction following illumina's protocol TrueSeq RNA Sample Preparation v2 Guide. The library was sequenced on an illumina HiSeq 2000/1000 instrument.

### Processing of sequencing reads

The quality of the sequence reads was assessed using fastQC version 0.9 (http://www.bioinformatics.babraham.ac.uk/projects/fastqc/). We observed a median phred score for each base between 30 and 40 across all samples. Ribosomal RNA contaminations were removed by mapping the 100bp single end reads to a collection of ribosomal RNA sequences using bowtie2 with standard parameters [16]. The fraction of rRNAs was between 53% and 63%. The remaining reads were mapped to the *Sus scrofa* genome assembly Ssc10.2/Ssr3 (http://hgdownload.cse.ucsc.edu/goldenPath/susScr3/bigZips/susScr3.fa.gz) using TopHat2 with default parameters [17]. Because the duplication rate was very high we decided to remove these duplicates using picard tools (http://picard.sourceforge.net), although this clearly underestimates the number of sequenced molecules. The high number of duplicates can be most probably attributed to the limited amount of input RNA available for the sequence library preparation, which required more PCR cycles than usual.

We combined the mapping files (bam files) of all samples for search of expressed regions. An expressed region is defined by a minimum length of 50 bp and a minimum average coverage of 5. These regions were combined with the preliminary annotation of the *Sus scrofa* genome and used to count reads with htseq_count (http://www-huber.embl.de/users/anders/HTSeq). Differential expression values were calculated using DESeq bioconductor library [18]. We compared the RNASeq results from two sperm RNA samples in each group of the supplemented and the control diet group for differential gene expression.

Similar to the microarray expression data we selected all genes that were differentially expressed in sperm cells between the two pairs of samples from the feeding experiment. We used transcripts that were differentially expressed on the nominal P>0.05 level between the groups and annotated them manually and used them to perform the enrichment analysis as described above.

The detailed results of the expression analysis in testes and sperm cells discussed in this publication have been deposited in NCBI's Gene Expression Omnibus and are accessible through GEO Series accession number GSE48778 (http://www.ncbi.nlm.nih.gov/geo/query/acc.cgi?&acc=GSE48778) [19].

## Supporting Information

Table S1 **Gene expression in testes of differentially fed boars.** List of differentially expressed genes in testes between 4 boars fed a control diet (control_diet) and 4 boars fed an experimental diet (suppl_diet).
(XLS)

Table S2 **Enrichment analysis report of differentially expressed genes in testes.** Differentially expressed genes in testes between the two diet groups were analyzed using the GeneGO Metacore pathway analysis software.
(XLS)

Table S3 **Enrichment analysis report of differentially expressed genes in boar sperm cells.** Differentially expressed genes in boar sperm cells between the two diet groups were analyzed using the GeneGO Metacore pathway analysis software.
(XLS)

Table S4 **DESeq analysis of differentially expressed genes in boar sperm cells.** Differentially expressed genes in boar sperm cells between 2 boars fed a control diet (9598/9600) and 2 boars fed an experimental diet (9597/9599) analyzed using the DESeq Bioconductor package.
(XLSX)
